# The mediating role of perceived social support on the relationship between lack of occupational coping self-efficacy and implicit absenteeism among intensive care unit nurses: a multicenter cross‑sectional study

**DOI:** 10.1186/s12913-024-11084-y

**Published:** 2024-05-21

**Authors:** Qin Lin, Mengxue Fu, Kun Sun, Linfeng Liu, Pei Chen, Ling Li, Yanping Niu, Jijun Wu

**Affiliations:** 1 Shulan International Medical College, Zhejiang Shuren University, Hangzhou, 310000 China; 2 Department of Rehabilitation, People’s Hospital of Jianyang, Jianyang, 641400 China; 3 Intensive Care Unit, West China Hospital, Sichuan University, Chengdu, 610044 China; 4 Department of Scientific Research, Sichuan Nursing Vocational College, Chengdu, 610100 China; 5 Department of Cardiology, People’s Hospital of Deyang, Deyang, 618099 China

**Keywords:** Perceived social support, Lack of occupational coping self-efficacy, Implicit absenteeism, Nurse, Intensive care unit, Management

## Abstract

**Background:**

Implicit absenteeism is very common among nurses. Poor perceived social support of intensive care unit nurses has a negative impact on their mental and physical health. There is evidence that lack of occupational coping self-efficacy can promote implicit absenteeism; however, the relationship between lack of occupational coping self-efficacy in perceived social support and implicit absenteeism of intensive care unit nurses is unclear. Therefore, this study aimed to evaluate the role of perceived social support between lack of occupational coping self-efficacy and implicit absenteeism of intensive care unit nurses, and to provide reliable evidence to the management of clinical nurses.

**Methods:**

A cross-sectional study of 517 intensive care unit nurses in 10 tertiary hospitals in Sichuan province, China was conducted, of which 474 were valid questionnaires with a valid recovery rate of 91.6%. The survey tools included the Chinese version of Implicit Absenteeism Scale, the Chinese version of Perceived Social Support Scale, the Chinese version of Occupational Coping Self-Efficacy Scale and the Sociodemographic characteristics. Descriptive analysis and Pearson correlation analysis were performed using SPSS version 22.0, while the mediating effects were performed using AMOS version 24.0.

**Results:**

The average of intensive care unit nurses had a total implicit absenteeism score of (16.87 ± 3.98), in this study, the median of intensive care unit nurses’ implicit absenteeism score was 17, there were 210 intensive care unit nurses with low implicit absenteeism (44.3%) and 264 ICU nurses with high implicit absenteeism (55.7%). A total perceived social support score of (62.87 ± 11.61), and a total lack of occupational coping self-efficacy score of (22.78 ± 5.98). The results of Pearson correlation analysis showed that implicit absenteeism was negatively correlated with perceived social support (*r* = -0.260, *P* < 0.001) and positively correlated with lack of occupational coping self-efficacy (*r* = 0.414, *P* < 0.001). In addition, we found that perceived social support plays a mediating role in lack of occupational coping self-efficacy and implicit absenteeism [*β* = 0.049, 95% *CI* of (0.002, 0.101)].

**Conclusions:**

Intensive care unit nurses had a high level of implicit absenteeism with a moderate level of perceived social support and lack of occupational coping self-efficacy. Nursing managers should pay attention to the nurses those who were within low levels of social support and negative coping strategies, and take measures to reduce intensive care unit nurses’ professional stress, minimize implicit absenteeism.

**Supplementary Information:**

The online version contains supplementary material available at 10.1186/s12913-024-11084-y.

## Background

Intensive care unit (ICU) nurses play a critical role in providing round-the-clock care to critically ill patients. However, the nature of their work can be stressful and demanding, resulting in physical and emotional challenges. The COVID-19 pandemic has further exacerbated these challenges, leading to negative outcomes for ICU nurses [1]. These challenges can take a toll on the physical and emotional well-being of ICU nurses, leading to negative outcomes such as low self-efficacy and implicit absenteeism [2]. The impact of the COVID-19 pandemic on the mental health of ICU nurses has been highlighted in recent research, with studies reporting high levels of anxiety, depression, and post-traumatic stress disorder among ICU nurses [3]. ICU nurses during the COVID-19 pandemic have been dealing with elevated levels of stress and emotional exhaustion [4]. The constant exposure to critically ill patients, the fear of personal infection, and the emotional toll of witnessing high mortality rates have contributed to increased psychological distress among ICU nurses [5]. Research suggests that during the COVID-19 pandemic, social support has become even more crucial for the mental health and well-being of healthcare professionals. A study found that perceived social support was inversely associated with anxiety and depression among healthcare workers during the pandemic [6]. In addition to the direct impact on mental health, the increased workload and exposure to infectious patients during the COVID-19 pandemic may exacerbate existing issues related to job satisfaction and burnout among ICU nurses. A study by Labrague and de Los Santos revealed that high levels of stress and workload significantly contributed to burnout among healthcare workers during the pandemic [7]. Besides, implicit absenteeism among ICU nurses poses a critical challenge within the broader framework of health systems and infrastructure, significantly affecting patient care, exacerbating workforce shortages, and contributing to systemic inefficiencies. ICU nurses are already in short supply, and implicit absenteeism contributes to workforce shortages. Emotionally disengaged nurses are more likely to experience burnout and turnover, leading to increased strain on the remaining staff and further exacerbating staffing shortages in critical care settings [8]. Implicit absenteeism can compromise patient safety and the quality of care provided in ICU. High levels of emotional disengagement may lead to decreased vigilance, diminished responsiveness to patient needs, and an increased likelihood of medical errors [9]. Therefore, it is important to explore strategies to support the mental health and well-being of ICU nurses during and after the COVID-19 pandemic.

Self-efficacy refers to an individual’s belief in their ability to perform a specific task or achieve a goal, and it plays a critical role in how individuals approach and cope with challenging situations. ICU nurses often face highly stressful and complex work environments, which characterized by high acuity patient care, time-sensitive decision-making, and emotional intensity. Firstly, the nature of ICU nursing involves caring for critically ill patients with complex medical conditions, ICU nurses are required to monitor vital signs, administer intricate treatments, and respond rapidly to dynamic patient situations [10]. This high acuity patient care demands a heightened level of attention and can lead to chronic stress and fatigue. Secondly, ICU nurses face constant time pressures and are often required to make swift, critical decisions, the need for quick and accurate responses to changing patient conditions adds a layer of stress to their work environment. This time-sensitive decision-making is inherent to ICU nursing and contributes to the overall complexity of their role [11]. Thirdly, ICU nurses regularly witness suffering, mortality, and family distress, the emotional intensity of providing care in life-threatening situations can lead to moral distress and emotional exhaustion. The burden of managing these emotions can have lasting effects on the mental health and well-being of ICU nurses [12]. Therefore, ICU nurse self-efficacy is an important area of research as it can affect job performance and job satisfaction. One study found that higher levels of self-efficacy were associated with greater job satisfaction and lower levels of emotional exhaustion among ICU nurses [13]. Another study suggested that ICU nurses with higher self-efficacy were more likely to engage in proactive coping behaviors, which in turn were associated with lower levels of emotional exhaustion [14].

Research has shown that social support is a critical factor in promoting the well-being of ICU nurses. Studies have found that social support from colleagues and supervisors is associated with lower levels of stress and burnout in ICU nurses [15]. In addition, social support from family and friends has been found to be important in buffering the negative effects of job stress on mental health in ICU nurses [16]. One study found that perceived social support from colleagues was positively associated with job satisfaction and negatively associated with emotional exhaustion among ICU nurses [17]. Another study found that social support from supervisors was positively associated with job satisfaction and negatively associated with turnover intention among ICU nurses [18]. Despite the recognized importance of social support for ICU nurses, more research is needed to fully understand the specific mechanisms through which social support operates and to develop effective interventions to support ICU nurses in the workplace.

Implicit absenteeism refers to a state where employees may be physically present at work but are emotionally or mentally disengaged, resulting in decreased job performance and overall contribution to the workplace [19]. While this term may not be widely used in the literature, the concept aligns with the broader understanding of presenteeism, which involves employees being on the job but not fully engaged or productive [20]. It is a more subtle form of employee disengagement than explicit absenteeism, such as calling in sick or taking time off. Implicit absenteeism is often associated with mental health conditions such as stress, burnout, and emotional exhaustion. Employees experiencing these mental health challenges may find it difficult to fully engage in their work, leading to a state of absenteeism despite being physically present [21]. Chronic health conditions can also contribute to implicit absenteeism, as employees dealing with physical health issues may struggle to fully engage in their tasks. This may manifest as reduced productivity, lack of focus, and an overall decline in job performance [22]. Implicit absenteeism can also impact nurses’ performance in the workplace, such as reduced patient interaction, reduced awareness of occupational protection, and lack of participation in professional development [23–25]. Research has suggested a negative relationship between nurse burnout, a concept closely related to implicit absenteeism, and job performance. For instance, a study by Van Bogaert et al. found that nurse burnout was significantly associated with lower perceived performance in various dimensions, including clinical care, teamwork, and job satisfaction [26]. One study found that high levels of job stress and low job control were associated with increased levels of implicit absenteeism among ICU nurses. The study suggested that interventions aimed at reducing job stress and increasing job control could be effective in reducing implicit absenteeism in this population [27]. Moreover, another study found that perceived organizational support was negatively associated with implicit absenteeism in ICU nurses. The study suggested that providing ICU nurses with a supportive work environment, such as opportunities for professional development and recognition, could reduce the occurrence of implicit absenteeism [28]. Moreover, a study on Chinese ICU nurses showed that implicit absenteeism were negatively correlated with perceived social support (*r*=-0.390, *P* < 0.05) and positively correlated with lack of occupational coping self-efficacy (*r* = 0.478, *P* < 0.05) [29]. The Job Demand-Resource (JD-R) Model is a theoretical framework in occupational and organizational psychology that was developed to understand the impact of job characteristics on employee well-being and performance. The model was initially proposed by Arnold Bakker and Evangelia Demerouti in the early 2000s [30]. The JD-R Model is widely used to investigate the factors that contribute to employee engagement, burnout, and overall job satisfaction. This model suggests that job resources, including social support, can buffer the impact of job demands on employee well-being and performance. Based on the above-mentioned literature reviews, this study puts forward the following hypotheses: First, lack of occupational coping self-efficacy is related to the implicit absenteeism of ICU nurses (H1). Second, lack of occupational coping self-efficacy is correlated with perceived social support of ICU nurses (H2). Third, perceived social support is correlated with the implicit absenteeism of ICU nurses (H3). Finally, perceived social support plays a mediating role in the relationship between lack of occupational coping self-efficacy, and implicit absenteeism (H4). By investigating the specific mechanisms through which social support operates, and the impact of the COVID-19 pandemic on ICU nurses, this study can provide important insights into interventions aimed at improving the well-being and job performance of ICU nurses.

## Methods

### Study design and ethics

A cross-sectional study was conducted in March 2022 using a convenience sampling method to select ICU nurses from 10 tertiary hospitals of 5 cities in Sichuan province, China. This study was conducted in accordance with the Helsinki Declaration. The study protocol was approved by the Ethics Committee of People’s Hospital of Deyang (2021-04-056-K01). The questionnaires remain anonymous, the first page of the online questionnaire is the informed consent form, participants indicate their agreement to participate in the survey by clicking the “Agree” option in the online assessment, all data collected are confidential and all participants had informed consent.

### Participants

A total of 517 questionnaires were issued and collected. 43 questionnaires were excluded due to evident patterns in the responses across various questionnaire items, and the surveys from the same hospital exhibited noticeable similarities. After eliminating 43 invalid questionnaires, 474 valid questionnaires were received, with a valid recovery rate of 91.6%.

The eligibility criteria were as follows: (1) registered nurses, (2) more than 12 months of ICU nursing experience, (3) willing to participate in the survey. The exclusion criteria were as follows: (1) training nurses or rotating nurses, (2) not working in the hospital during the survey period, such as long-term sick leave or maternity leave.

### Survey tools

#### Sociodemographic characteristics

Sociodemographic characteristics included age, gender, marital status, educational background, professional title, management position, working experience in ICU, employment form, turnover intention, physical pain, occupational stress, night shift experience and workplace violence.

#### The Chinese version of implicit absenteeism scale

The scale was developed by Koopman et al. [31] and translated and revised in to Chinese by Zhao Fang [32]. First, Zhao Fang and others translated, back-translated, and culturally adapted the scale. Subsequently, they conducted a survey on 935 staff members to validate the reliability and validity of the scale. The results indicated that the Cronbach’s α coefficients for each dimension of the scale ranged from 0.76 to 0.90. The structural validity revealed two latent factors, namely, work constraints and work vigor, with a cumulative variance contribution rate of 81.01%. The reliability and validity of the scale were deemed satisfactory. In another study, Liu Jia-wen et al. employed the same scale to conduct a questionnaire survey on 150 emergency department nurses in Nanchang, China, from September to October 2020 [33]. The Cronbach’s α coefficient for the scale was found to be 0.71, indicating good reliability. This scale consists of 6 items and the scale is used to estimate the employee productivity loss caused by the implicit absenteeism with a specific health status in the past month. This scale is based on a 5-point Likert scale, ranging from “completely disagree” to “completely agree”. The score for each item ranges from 1 to 5, and the total score ranges from 6 to 30, with higher scores indicating higher levels of productivity loss due to health status and the less effective attendance.

#### The Chinese version of perceived social support scale

The scale was developed by Zimet et al. [34] and translated and revised in to Chinese by Jiang Qian-jin [35]. In December 2019, Xiang Feng-ming and others employed this scale to conduct a questionnaire survey on 182 novice nurses in Wenzhou, China. The results showed that the Cronbach’s α coefficients for the overall scale and each dimension were 0.856, 0.803, 0.851, and 0.866, respectively [36]. The scale was used to measure perceived social support of ICU nurses. This scale consists of 3 dimensions and 12 items: 4 items for family support, 4 items for friends support, 4 items for other support. This scale is based on a 7-point Likert scale, 1 point stand for very disagree and 7 points stand for very agree. The total score ranges from 12 to 84, with higher scores indicating higher levels of perceived social support. The Cronbach’s alpha coefficient of this scale 0.90.

#### The Chinese version of occupational coping self-efficacy scale

The Chinese version of Occupational Coping Self-Efficacy Scale was used to measure the lack of occupational coping self-efficacy of ICU nurses. The scale was developed by Pisanti et al. [37] and translated and revised in to Chinese by Zhai Yan-xue [38]. First, Zhai Yan-xue organized researchers to translate and back-translate the scale. Subsequently, cultural adaptation was conducted by three experienced experts. Following this, modifications were made based on a preliminary survey of 50 nurses. Finally, a survey was conducted on 1172 nurses from five public hospitals to validate the reliability and validity of the scale. The results indicated a Cronbach’s α coefficient of 0.882, a test-retest reliability of 0.991, I-CVI of 0.833 ∼ 1.000, S-CVI/UA of 0.889 and S-CVI/Ave of 0.981, demonstrating good reliability and validity of the scale [38]. This scale consists of 2 dimensions and 9 items: 6 items for professional burden, 3 items for difficulties in getting along with each other. This scale is based on a 5-point Likert scale, 1 point means “strongly disagree”, 5 points means “strongly agree”, and the total score ranges from 9 to 45, with higher scores indicating lower levels of occupational coping self-efficacy, means that the lack of occupational coping self-efficacy. The Cronbach’s alpha coefficient of this scale was 0.88, and the Cronbach’s alpha coefficients of the subscales were 0.79 and 0.87.

### Data collection

We contacted the head nurses of ICU departments in 10 tertiary hospitals of 5 cities in Sichuan province, and distributed the online links of questionnaires to them to finish the survey. Voluntary and anonymity principle, inclusion and exclusion criteria were indicated on the first page of the online questionnaire. If ICU nurses clicked on the online link and submitted the questionnaire, it was informed consent by default. We set all answers must be filled out before submission. And two researchers checked the questionnaires to ensure the validity and integrity of the survey.

### Data analysis

This study used SPSS version 22.0 and AMOS version 24.0 (IBM, Armonk, NY, USA) for statistical analysis of the data. Firstly, descriptive analysis was used to describe the sociodemographic characteristics and main variables of ICU nurses. Count data were expressed as percentage (%). Measurement data were expressed as (mean ± standard deviation), in addition, independent samples t-test and one-way ANOVA were used for comparison between groups. Pearson correlation analysis was performed to analysis the correlation of social support, lack of occupational coping self-efficacy and implicit absenteeism. Besides, we used lack of occupational coping self-efficacy as the independent variable and implicit absenteeism as the dependent variable to examine the mediating role of social support. In this study, we take α = 0.05 as the test standard.

## Results

### Sociodemographic characteristics of ICU nurses

As detailed in Table 1, a total of 474 ICU nurses were included in this study with a mean age of 32.19 years (range 21 to 56) and a mean year of ICU working experience of 8.07 years (range 1 to 36). Most of the ICU nurses were female (91.8%) and married (58.0%). In terms of educational background, 77.8% of ICU nurses have a bachelor’s degree. Besides, 82.7% of ICU nurses were in the authorized strength and contract system. Nearly 18% of ICU nurses were are experiencing physical pain. About 35% of ICU nurses admitted that they have high level occupational stress. In addition, 34.5% of ICU nurses have experienced workplace violence.

There were significant differences in the marital status, professional title, management position, working experience in ICU, turnover intention, physical pain, occupational stress, night shift experience and workplace violence between ICU nurses with high and low implicit absenteeism (all *P* < 0.05). And no significant differences were found in the in the gender, age, educational background and employment form between ICU nurses with high and low implicit absenteeism (all *P* > 0.05).

**Table 1:** Sociodemographic characteristics of ICU nurses (*N* = 474)

Characteristics	*n*	%	Mean ± SD	t/F	*P*
Gender				-0.881	0.379
Male	39	8.2%	16.33 ± 3.60		
Female	435	91.8%	16.92 ± 4.01		
Age (years)				1.166	0.322
<30	172	36.3%	16.79 ± 3.94		
30∼<40	255	53.8%	17.10 ± 3.95		
40∼<50	36	7.6%	15.94 ± 4.73		
≥ 50	11	2.3%	15.91 ± 1.30		
Marital status				4.702	0.003
Unmarried	136	28.7%	16.63 ± 3.96		
Married	275	58.1%	16.84 ± 3.98		
Divorced	52	10.9%	18.31 ± 3.75		
Other	11	2.3%	13.82 ± 2.92		
Educational background				0.817	0.443
College degree and below	93	19.6%	16.69 ± 3.95		
Undergraduate degree	369	77.8%	16.96 ± 4.01		
Master degree and above	12	2.6%	15.58 ± 2.96		
Professional title				3.006	0.030
Nurse	74	15.6%	17.16 ± 3.74		
Primary nurse	234	49.4%	16.78 ± 4.08		
Senior nurse	145	30.6%	17.21 ± 3.72		
Supervisor nurse	21	4.5%	14.52 ± 4.65		
Management position				4.637	0.010
Clinical nurse	382	80.6%	16.88 ± 3.95		
Group leader nurse	63	13.3%	17.70 ± 3.74		
Head nurse	29	6.1%	15.00 ± 4.25		
Working experience in ICU (years)				3.107	0.046
<5	170	35.9%	16.31 ± 4.05		
5∼<10	165	34.8%	17.38 ± 4.04		
≥ 10	139	29.3%	16.96 ± 3.75		
Employment form				-0.776	0.438
Authorized strength	392	82.7%	16.94 ± 3.83		
Other	82	17.3%	16.56 ± 4.64		
Turnover intention*				31.052	0.000
Low	265	55.9%	15.82 ± 3.72		
Medium	178	37.6%	17.78 ± 3.90		
High	31	6.5%	20.61 ± 3.01		
Physical pain				4.676	0.000
Yes	83	17.5%	18.69 ± 3.28		
No	391	82.5%	16.49 ± 4.01		
Occupational stress				9.714	0.000
Low	58	12.3%	15.55 ± 3.77		
Medium	249	52.5%	16.51 ± 3.88		
High	167	35.2%	17.86 ± 3.99		
Night shift experience				2.314	0.021
Yes	398	84.0%	17.06 ± 3.91		
No	76	16.0%	15.91 ± 4.20		
Workplace violence				5.450	0.000
Yes	164	34.6%	18.20 ± 3.95		
No	310	65.4%	16.17 ± 3.81		

*Note* * When the respondent selects ‘low’ it indicates that he/she is highly unlikely to resign. When the respondent selects ‘medium’ it suggests that he/she often hesitates about whether to resign. When the respondent selects ‘high’ it signifies that he/she is very likely to resign

### Scores of implicit absenteeism scale, perceived social support scale and occupational coping self-efficacy scale

As shown in Table 2, the average of ICU nurses had a total implicit absenteeism score of (16.87 ± 3.98), indicating that ICU nurses had a high level of implicit absenteeism.

Previous research [39] has reported that more than half of nurses have implicit absenteeism and take the median of nurses’ implicit absenteeism score as the cut-off point to differentiate the high and low implicit absenteeism. In this study, the median of ICU nurses’ implicit absenteeism score was 17. Therefore, there were 210 ICU nurses with low implicit absenteeism (44.3%) and 264 ICU nurses with high implicit absenteeism (55.7%).

In addition, a total perceived social support score of (62.87 ± 11.61), indicating that ICU nurses had a moderate level of perceived social support, and a total lack of occupational coping self-efficacy score of (22.78 ± 5.98), indicating that ICU nurses had a moderate level of lack of occupational coping self-efficacy. The detailed information is shown in Table 3.

**Table 2:** Mean and standard deviation of each item in implicit absenteeism scale (*N* = 474)

Items	Min	Max	Mean	Standard deviation
Q1 In the past month, due to health problems, my work pressure has become more difficult to adjust.	1	5	2.95	1.09
Q2 In the past month, due to health problems, I was unable to complete the difficult tasks at work.	1	5	2.50	1.07
Q3 In the past month, due to health problems, I couldn’t get pleasure from my work.	1	5	2.79	1.15
Q4 In the past month, due to health problems, I felt it was impossible to carry out some work tasks.	1	5	2.59	1.13
Q5 In the past month, despite my health problems, I was able to concentrate on finishing my work.*	1	5	2.90	1.12
Q6 In the past month, despite my health problems, I still feel energetic and can finish all my work.*	1	5	3.13	1.06
**Implicit absenteeism**	6	28	16.87	3.98

*Note* * The item was inverse-coded

**Table 3:** Mean and standard deviation of each variable (*N* = 474)

Variables	Number of Items	Min	Max	Mean	Standard deviation
**Perceived social support**	12	15	84	62.87	11.61
Family support	4	4	28	20.98	4.86
Friends support	4	4	28	20.76	4.15
Other support	4	7	28	21.12	3.90
**Lack of Occupational coping self-efficacy**	9	9	45	22.78	5.98
Professional burden	6	6	30	15.84	3.97
Difficulties in getting along with each other	3	3	15	6.93	2.42

### Analysis of the correlation between implicit absenteeism, perceived social support and lack of occupational coping self-efficacy

The results of Pearson correlation analysis showed that implicit absenteeism was negatively correlated with perceived social support (*r* = -0.260, *P* < 0.001) and positively correlated with lack of occupational coping self-efficacy (*r* = 0.414, *P* < 0.001), Table 4.

**Table 4:** Correlations between implicit absenteeism, perceived social support and lack of occupational coping self-efficacy of ICU nurses (*N* = 474)

Variables		1	2	3	4	5	6	7	8
**Implicit absenteeism**	1. total score	1.000							
**Perceived social support**	2. total score	-0.260**	1.000						
	3. family support	-0.229**	0.897**	1.000					
	4. friends support	-0.212**	0.902**	0.689**	1.000				
	5. other support	-0.261**	0.896**	0.688**	0.762**	1.000			
**Lack of Occupational coping self-efficacy**	6. total score	0.414**	-0.348**	-0.306**	-0.299**	-0.337**	1.000		
	7. professional burden	0.379**	-0.304**	-0.285**	-0.253**	-0.280**	0.961**	1.000	
	8. difficulties in getting along with each other	0.398**	-0.361**	-0.288**	-0.323**	-0.371**	0.890**	0.729**	1.000

** At the 0.01 level (double tail), the correlation is significant

### Mediating effect of perceived social support between lack of occupational coping self-efficacy and implicit absenteeism in ICU nurses

**Fig. 1:**
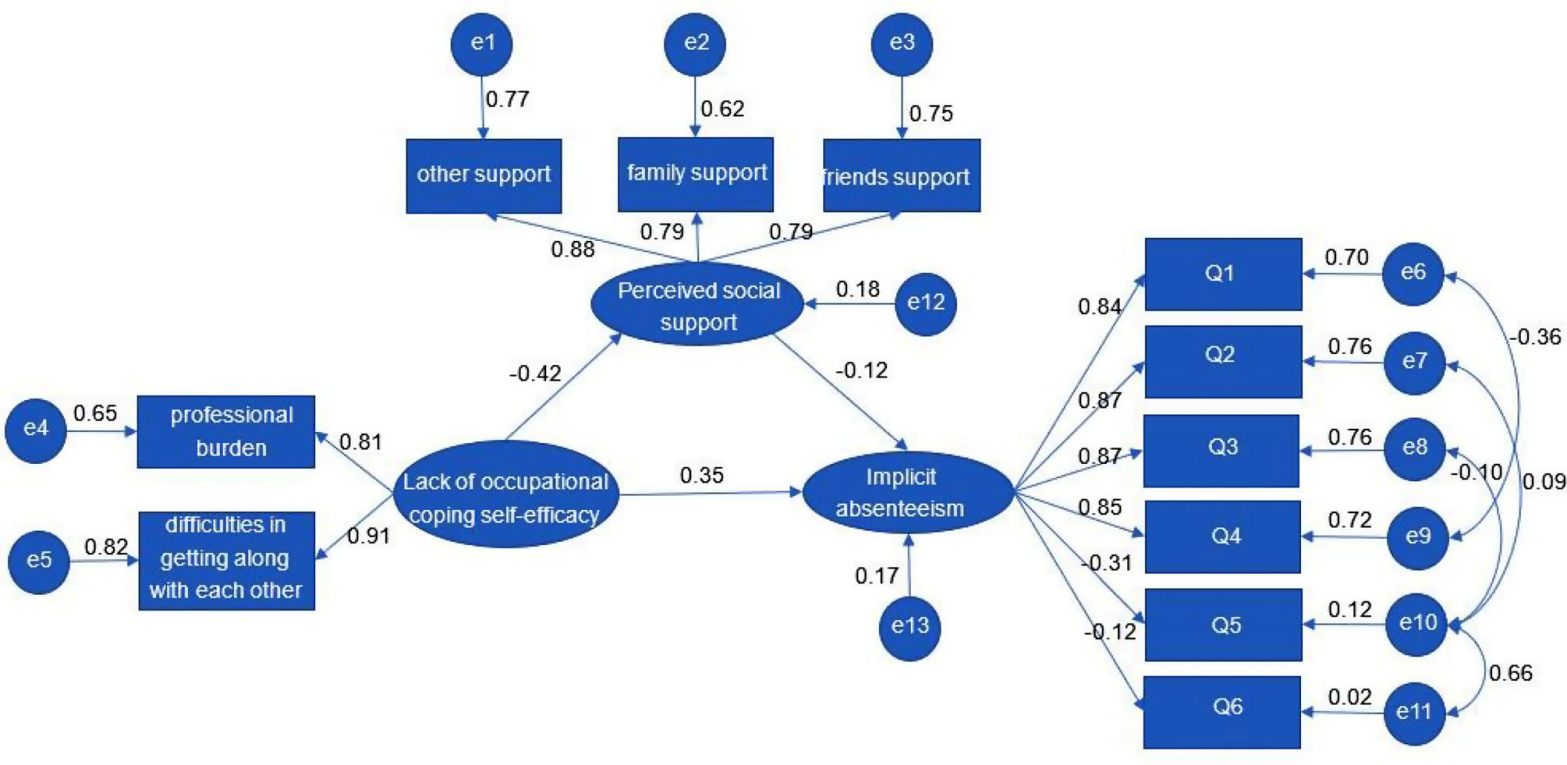
The mediating effect model of perceived social support between lack of occupational coping self-efficacy and implicit absenteeism in ICU nurses

Figure 1; Table 5 show the Structural Equation Model results. The standardized model paths are shown in Fig. 1. The direct effect, indirect effect, and total effect are shown in Table 5. The standardized model had a good model fit with the data (Table 6).

**Table 5:** Effects of perceived social support and lack of occupational coping self-efficacy on implicit absenteeism

Path	Standardized effect (β)	S.E.	*P*	Bootstrap 95%CI		Effect ratio (%)
				Lower	Upper	
Direct effect Lack of occupational coping self-efficacy → Implicit absenteeism	0.355	0.058	0.001	0.236	0.461	87.87
Indirect effect Lack of occupational coping self-efficacy → Perceived social support → Implicit absenteeism	0.049	0.025	0.041	0.002	0.101	12.13
Total effect Lack of occupational coping self-efficacy → Implicit absenteeism	0.404	0.047	0.000	0.304	0.490	

As shown in Table 5, lack of occupational coping self-efficacy (*β* = 0.404, for total effect) had significant positive relationships with implicit absenteeism. The results also indicated that the indirect effect (*β* = 0.049) of lack of occupational coping self-efficacy on implicit absenteeism was significant as well as its direct effect on perceived social support (*β* = -0.420). These findings show that higher occupational coping self-efficacy was related to lower implicit absenteeism and higher perceived social support, and that perceived social support partially mediated the relationship between lack of occupational coping self-efficacy and implicit absenteeism due to significant direct and indirect paths.

**Table 6:** Model fit indices

Index	χ^2^	d.f.	χ^2^/ d.f.	RMSEA	GFI	AGFI	NFI	RFI	IFI	TLI	CFI
Research Model	75.649	37	2.045	0.047	0.971	0.949	0.975	0.963	0.987	0.981	0.987
criteria			<3	<0.05	>0.9	>0.9	>0.9	>0.9	>0.9	>0.9	>0.9

## Discussion

Nursing workforce is critical in delivering quality care to patients in healthcare settings. However, absenteeism among nurses has become a significant concern for healthcare organizations globally. The phenomenon of nurse’s implicit absenteeism has been extensively studied in both China and other countries. A study found that the prevalence of nurse’s implicit absenteeism was 11.7% in China, and the factors influencing implicit absenteeism included age, working experience, and job satisfaction [40]. Similarly, a study conducted in Saudi Arabia reported that 24.7% of nurses experienced implicit absenteeism, with workload and job stress being the significant predictors [41]. Moreover, research conducted in the United States (US) and Europe also highlighted the problem of implicit absenteeism among nurses. A study in the US found that implicit absenteeism was associated with burnout, job dissatisfaction, and intention to leave the profession [42]. In addition, a study reported that implicit absenteeism was associated with workload, job demands, and role ambiguity [43]. Our study showed that the implicit absenteeism score of ICU nurses was (16.87 ± 3.98), which was similar to implicit absenteeism score of (17.25 ± 3.51) for ICU nurses in another study in China [44]. Similar research conclusions are also found in 200 ICU nurses from tertiary comprehensive hospitals in Beijing, where their implicit absenteeism score was (16.65 ± 4.69) [45]. This implies that ICU nurses in China generally experience implicit absenteeism, a result deserving attention from nursing managers. Analyzing this, it may be attributed to the global shortage of nursing personnel, where ICU nurses face a similar shortage of human resources. Given that ICU patients are often critically ill with rapidly changing conditions, the tasks of ICU nurses are less substitutable, making them prone to implicit absenteeism. Additionally, in the ICU team nursing model, team members taking sick leave may increase the workload for other members. Based on a sense of responsibility to colleagues, many tend to choose implicit absenteeism. Hence, healthcare organizations must develop strategies to manage and reduce implicit absenteeism among nurses to ensure quality patient care.

In recent years, research on perceived social support among ICU nurses has gained attention both in China and abroad. Perceived social support is the belief that one has access to individuals, groups, or networks that provide assistance and care in times of need [46]. ICU nurses are exposed to various stressors such as heavy workloads, long working hours, and critically ill patients, which can lead to emotional exhaustion, burnout, and turnover intention [47]. Therefore, perceived social support is crucial for their psychological well-being and job satisfaction. In China, several studies have investigated perceived social support among ICU nurses. For example, a study found that ICU nurses perceived low social support from their colleagues and supervisors, which was negatively associated with their job satisfaction [48]. Another study showed that ICU nurses who perceived high social support had lower levels of burnout and higher levels of work engagement [49]. Similarly, a study conducted in the Netherlands revealed that ICU nurses who perceived high social support from their colleagues had lower levels of emotional exhaustion and turnover intention [50]. Another study found that perceived social support from family and friends was positively associated with job satisfaction and negatively associated with emotional exhaustion among ICU nurses [51]. In this study, the perceived social support score of ICU nurses was (62.87 ± 11.61) points, which is lower than that of the research of Wu Peng [52] and Wang Li-jiao [53], indicating that nurses’ perceived social support had a moderate level in China. This suggests that the level of social support among ICU nurses in China is generally low. Previous research suggests a close correlation between nurses’ professional identity, job performance, and social support levels. Nurses who receive good support from family, friends, and other social networks often can maintain both physical and mental health, smoothly handle job responsibilities, accomplish tasks successfully, and experience higher job satisfaction [52, 54]. With nurses being recognized as a high-risk profession, the social support received by nurses has been widely studied in the field of nursing human resources management. Concerningly, the level of social support for nurses in China and globally is not high. Our nurses are currently experiencing significant work pressure and physical fatigue, making it challenging for them to fully engage in their work. Therefore, it is important for healthcare organizations to develop interventions that enhance social support among ICU nurses to promote their well-being and reduce turnover intention. Overall, it is important for healthcare organizations to develop interventions that enhance social support among ICU nurses to promote their well-being and reduce turnover intention.

Occupational coping self-efficacy refers to an individual’s belief in their ability to effectively manage job-related stressors and challenges [55]. It is a crucial factor for promoting nurses’ occupational growth and sense of occupational benefit, as well as improving patient outcomes. Studies have shown that ICU nurses have relatively low levels of occupational coping self-efficacy. For example, a study found that ICU nurses with higher levels of occupational coping self-efficacy reported lower levels of emotional exhaustion and depersonalization, and higher levels of personal accomplishment [56]. A study also found that occupational coping self-efficacy was positively associated with job satisfaction among ICU nurses in China [57]. Another study found that occupational coping self-efficacy was positively associated with job satisfaction and negatively associated with burnout among ICU nurses in Taiwan [58]. In addition, a study showed that ICU nurses in Saudi Arabia with higher levels of occupational coping self-efficacy reported lower levels of emotional exhaustion and higher levels of personal accomplishment [59].

The results of this study suggest that the total score of lack of occupational coping self-efficacy was (22.78 ± 5.98). This result suggests that the self-efficacy levels of the majority of ICU nurses in China need improvement. The findings of this study are consistent with a previous survey on the self-efficacy of ICU nurses in China [60], and compared to other clinical nurses in China, ICU nurses exhibit lower levels of self-efficacy [61–62]. Additionally, when compared to professions such as secondary school teachers and pilots [63–64], ICU nurses seem to experience more widespread lower self-efficacy levels. In China, most ICU nurses work an average of more than 8 h per day. Under the prolonged work pressures, ICU nurses have less time available for interpersonal relationships, family activities, rest, and sleep. Therefore, this may lead to occupational burnout, lower self-efficacy among ICU nurses, and potentially trigger work-family conflicts. The above results provide clear evidence indicating that nursing managers should pay closer attention to the self-efficacy levels of ICU nurses in the future, as it is closely associated with the professional development of ICU nurses.

Our correlation results show that implicit absenteeism of ICU nurses was negatively correlated with perceived social support and its various dimensions (*r*=-0.212∼-0.260, *P*<0.01). The lower the perceived social support, the higher the implicit absenteeism. The results of this study indicate a negative correlation between support from family and implicit absenteeism among ICU nurses, consistent with previous research [65]. Family support not only ensures the maintenance of a positive mood for ICU nurses but also contributes to their physical well-being and active engagement in work. Furthermore, support from friends is negatively correlated with implicit absenteeism among ICU nurses. Friends support allow ICU nurses to feel embraced, understood, and assisted from the outside, enabling them to confront life and work challenges positively and facilitating the completion of work tasks [66]. Lastly, support from other sources is negatively correlated with implicit absenteeism among ICU nurses. According to reports [29], having more organizational support enables nurses to better immerse themselves in their work, where their personal values are fully reflected in patient care. This leads to a more positive response to work and reduces the impact of compromised health productivity to a lower level. In contrast, implicit absenteeism of ICU nurses was positively correlated with lack of occupational coping self-efficacy and its various dimensions (*r* = 0.379 ∼ 0.414, *P*<0.01). The higher the occupational coping self-efficacy, the lower the implicit absenteeism. These findings suggest that both perceived social support and lack of occupational coping self-efficacy are important factors in predicting implicit absenteeism among ICU nurses. Given the findings, it is important for healthcare organizations to prioritize interventions that can help to increase perceived social support and occupational coping self-efficacy among ICU nurses. For example, providing opportunities for social support, such as peer mentoring or support groups, can help to reduce feelings of isolation and increase job satisfaction [67]. Additionally, training programs that focus on developing coping skills and self-efficacy can help nurses to better manage the demands of their job and feel more confident in their abilities [68]. Here are some feasible approaches that healthcare organizations can consider to improve ICU nurses’ perceived social support. Firstly, establishing peer mentoring programs where experienced ICU nurses provide support and guidance to newer or less experienced colleagues. Encouraging regular interactions and check-ins to foster a sense of camaraderie and mutual support. secondly, creating support groups within the ICU setting, where nurses can share experiences, discuss challenges, and provide emotional support to each other. Facilitating group discussions led by mental health professionals to address common stressors and coping strategies. Thirdly, providing training on effective communication skills to enhance interpersonal relationships among ICU team members. Emphasizing active listening and empathy to create a supportive environment. The above strategies for social support are among colleagues within the ICU setting. ICU nurses’ social support from family and friends are different with colleagues support and may implement distinct strategies recognizing the unique dynamics of each relationship. For support from family and friends, interventions could involve educational sessions for family and friends to understand the demands and stressors specific to ICU nursing, encouraging open communication channels between nurses and their loved ones, and providing resources for family support. This might include counseling services or support groups for family and friends of ICU nurses. In addition, the healthcare organizations can improve the occupational coping self-efficacy of ICU nurses through the following measures. First of all, conducting workshops focused on enhancing specific coping skills, such as time management, stress reduction techniques, and conflict resolution. Encouraging ongoing professional development to build a sense of competence. Besides, implementing recognition programs to acknowledge the hard work and dedication of ICU nurses. Providing regular feedback and appreciation for their contributions to patient care. Finally, organizing team-building activities to foster a positive work environment and strengthen teamwork among ICU staff. Creating a culture that values collaboration and mutual support. Furthermore, the relationship between social support, self-efficacy, and absenteeism due to physical health is a complex interplay influenced by various factors. On the one hand, social support, whether from colleagues, friends, or family, can act as a buffer against ICU nurses’ stress. Lower stress levels are associated with improved physical health and a reduced likelihood of needing time off due to health issues. On the other hand, social support networks can provide practical assistance, such as help with ICU nurses’ childcare or transportation, which may mitigate the impact of physical health issues on absenteeism. Having a reliable support system may reduce the need for extended time off for health-related issues. Self-efficacy is associated with absenteeism due to physical health, which is reflected in the following aspects. First, high self-efficacy is associated with a greater likelihood of adopting and maintaining healthy behaviors, such as regular exercise and a balanced diet. Healthy lifestyles contribute to overall well-being and can reduce ICU nurses’ risk of absenteeism due to physical health issues. next, individuals with high self-efficacy often possess effective coping mechanisms to manage pain, discomfort, or chronic conditions. The ability of ICU nurses’ to cope with health challenges may reduce the severity and duration of illnesses, potentially minimizing absenteeism.

The results of our mediating role analysis show that ICU nurses’ the lack of occupational coping self-efficacy had a direct positive predictive influence on their implicit absenteeism and that perceived social support had a partial mediating effect between lack of occupational coping self-efficacy and implicit absenteeism. The higher the level of occupational coping self-efficacy, the better the perceived social support and the lower the implicit absenteeism. The reasons for this relationship may be as follows: firstly, as the level of perceived social support increases, ICU nurses are more likely to feel external support from family, friends, and other sources, which can be utilized to effectively solve their problems or difficulties [69]. Secondly, higher levels of perceived social support can help ICU nurses to redefine difficult situations and strengthen their ability to regulate feelings of distrust, anxiety, and fear, thereby enhancing their positive attitudes towards handling difficulties and reducing implicit absenteeism [70]. In addition, a higher level of occupational self-efficacy enables ICU nurses to have a correct understanding of the challenges they face at work, form positive professional identities and values, and increase their confidence in dealing with difficulties [71]. In the practice environments of many hospitals in China, issues such as workload overload, poor working conditions, and lack of adequate compensation may exist [72]. The working environment in the ICU is often characterized by its enclosed nature, and the workload is frequently higher than in other departments. This may explain why many ICU nurses may develop lower self-efficacy and lack social support. Social support has the potential to uplift the work enthusiasm and professional spirit of ICU nurses, fostering a positive professional attitude and encouraging them to contribute to patient health and the development of medical institutions. However, as observed in this study, when experiencing lower self-efficacy, ICU nurses exhibit compromised health productivity, refrain from making additional efforts for the medical institution, and develop a negative attitude towards their work. If the social support for ICU nurses diminishes due to lower self-efficacy, it is unsurprising that their sense of mission as “health guardians” diminishes as well, meaning ICU nurses may no longer see serving the health of patients as their mission. As mentioned in the Conservation of Resources theory, when ICU nurses lose their resources (similar to losing social support in this study), they may experience varying degrees of psychological stress and lose confidence in the organization. With increased separation, psychological or physical issues may arise, such as ICU nurses being physically unwilling to engage in work, emotionally reluctant to integrate into the work team, cognitively becoming less active, and concealing their feelings and thoughts, all of which signify the occurrence of implicit absenteeism. Moreover, it can further impact the attitudes, behaviors, and beliefs of ICU nurses towards their profession, negatively affecting professional identity and a sense of professional mission. Therefore, hospital nursing managers need to pay closer attention to the current status of self-efficacy and social support among ICU nurses, strengthening their professional attitudes from aspects of organizational support, colleague support, and material support to promote healthy practices in their profession.

On November 29th, 2021, the General Office of the People’s Government of Sichuan Province issued the Implementation Plan for Promoting the High-quality Development of Public Hospitals in Sichuan Province, which emphasized the reform of personnel management system and the increase of nurses’ equipment, so that the overall ratio of doctors to nurses in public hospitals gradually reached about 1: 2. In addition, the salary distribution system should be reformed to encourage the internal distribution of hospitals to be tilted towards high-risk and high-intensity posts. The above policies and programs provide strong external social support for ICU nurses. Identifying institutional gaps and proposing improvements to support the workforce involves a comprehensive examination of existing policies, practices, and organizational structures. Common gaps include limited mental health support with suggestions for comprehensive programs and manager training, inadequate work-life balance policies calling for flexible arrangements and clear remote work options, a lack of professional development opportunities necessitating ongoing training, mentorship programs, and support for continuous learning, and insufficient diversity and inclusion initiatives requiring diversity training, committees, and regular assessments. Other gaps encompass poor communication channels, suggesting enhancements to internal communication strategies and regular town hall meetings, limited health and wellness programs requiring initiatives addressing physical and mental health, and inadequate remote work infrastructure, calling for technology investment and clear policies. Additionally, the absence of recognition and rewards programs underscores the need for implementation alongside leadership training, regular employee surveys, clear career progression paths, encouragement of peer support networks, and continuous evaluation and adaptation of workplace policies for ongoing improvement. Addressing these gaps and implementing improvements necessitates a collaborative effort from leadership, human resources, and employees, emphasizing ongoing assessment, communication, and a genuine commitment to workforce well-being and development.

### Limitations and recommendations

This research has some limitations that need to be acknowledged. Firstly, this study is a cross-sectional design, which cannot determine the causal relationship between variables.

Secondly, this study employed a convenience sampling method to select participants, which may result in meaningful differences in various sociodemographic categories. These differences among the population could limit the generalizability of the results to a broader population, introduce bias to the sample, and potentially act as confounding variables affecting the relationship between the independent and dependent variables. Therefore, in future research, efforts to enhance the representativeness of the sample will be a focus of our endeavors. Finally, this study was conducted during the COVID-19 pandemic, which may have led to higher levels of implicit absenteeism and lower levels of occupational coping self-efficacy being measured.

Despite these limitations, this study can provide valuable information for future research. For example, this study describes the occurrence of implicit absenteeism among ICU nurses from the perspectives of social support and self-efficacy and establishes a structural equation model, making the study of implicit absenteeism more comprehensive and richer. Additionally, this study confirms the mediating role of perceived social support in the relationship between lack of occupational coping self-efficacy and implicit absenteeism among ICU nurses, suggesting that increasing perceived social support and occupational coping self-efficacy can reduce implicit absenteeism, decrease negative emotions and psychological stress, improve ICU nurses’ job satisfaction and promote their mental health.

## Conclusions

The results of this study indicate that there is a high prevalence of implicit absenteeism among ICU nurses, with 55.7% of ICU nurses evaluated as having a high level of implicit absenteeism and 44.3% evaluated as having a low level of implicit absenteeism. In addition, implicit absenteeism among ICU nurses is negatively correlated with perceived social support and positively correlated with lack of occupational coping self-efficacy. Perceived social support plays a significant mediating role between lack of occupational coping self-efficacy and implicit absenteeism among ICU nurses. In future departmental management, ICU managers need to pay attention to nurses with low levels of social support and negative coping strategies, and take measures such as providing peer support, forming work groups, and arranging work tasks reasonably to reduce nurses’ professional stress, minimize implicit absenteeism, and promote the development of high-quality nursing teams.

### Electronic supplementary material

Below is the link to the electronic supplementary material.

Supplementary Material 1

## Data Availability

All data generated or analyzed during this study are included in this published article.
