# Supplementary material for: The mediating role of perceived social support on the relationship between lack of occupational coping self-efficacy and implicit absenteeism among intensive care unit nurses: a multicenter cross‑sectional study
Source: BMC Health Serv Res. 2024 May 21;24:653. doi: 10.1186/s12913-024-11084-y (PMC11110179; doi:10.1186/s12913-024-11084-y)
Supplement: Supplementary file 1 — Supplementary Material 1 [file 12913_2024_11084_MOESM1_ESM.docx]

**The three scales used in the study**

**1. Implicit A****bsenteeism Scale**

Evaluate each statement based on the following rating scale. Place a "√" in the corresponding box that best represents your actual situation. Utilize a 5-level scoring system, ranging from completely disagree to completely agree for each option.

| **Items** | completely disagree | Somewhat Disagree | Neutral | Somewhat Agree | completely agree |
| --- | --- | --- | --- | --- | --- |
| In the past month, due to health problems, my work pressure has become more difficult to adjust. |  |  |  |  |  |
| In the past month, due to health problems, I was unable to complete the difficult tasks at work. |  |  |  |  |  |
| In the past month, due to health problems, I couldn't get pleasure from my work. |  |  |  |  |  |
| In the past month, due to health problems, I felt it was impossible to carry out some work tasks. |  |  |  |  |  |
| In the past month, despite my health problems, I was able to concentrate on finishing my work. |  |  |  |  |  |
| In the past month, despite my health problems, I still feel energetic and can finish all my work. |  |  |  |  |  |

**2. Perceived Social Support Scale**

According to the following rating scale, place a '√' in the corresponding box that best represents your actual situation. Utilize a 7-level scoring system, ranging from strongly disagree to strongly agree for each option.

| **Items** | Strongly Disagree | Disagree | Somewhat Disagree | Neutral | Somewhat Agree | Agree | Strongly agree |
| --- | --- | --- | --- | --- | --- | --- | --- |
| When I encounter problems, some people (colleagues, friends, relatives) are there for me. |  |  |  |  |  |  |  |
| I can share joys and sorrows with some people (colleagues, friends, relatives). |  |  |  |  |  |  |  |
| When I face difficulties, some people (colleagues, friends, relatives) are a true source of comfort for me.  There are certain people (colleagues, friends, relatives) in my life who care about my feelings. |  |  |  |  |  |  |  |
| My family can provide practical help for me. |  |  |  |  |  |  |  |
| When needed, I can receive emotional assistance and support from my family. |  |  |  |  |  |  |  |
| I can discuss my problems with my family. |  |  |  |  |  |  |  |
| My family is willing to assist me in making various decisions. |  |  |  |  |  |  |  |
| My friends can genuinely help me. |  |  |  |  |  |  |  |
| I can rely on my friends when facing difficulties. |  |  |  |  |  |  |  |
| My friends can share joys and sorrows with me. |  |  |  |  |  |  |  |
| I can discuss my problems with my friends. |  |  |  |  |  |  |  |

**3. Occupational Coping Self-Efficacy Scale**

According to the following rating scale, place a '√' in the corresponding box that best represents your actual situation. Utilize a 5-level scoring system, ranging from strongly disagree to strongly agree for each option.

| **Items** | Strongly Disagree | Somewhat Disagree | Neutral | Somewhat Agree | Strongly agree |
| --- | --- | --- | --- | --- | --- |
| Difficulty interacting with patients |  |  |  |  |  |
| Juggling multiple tasks simultaneously |  |  |  |  |  |
| Difficulty dealing with patients' families |  |  |  |  |  |
| Unclear work procedures |  |  |  |  |  |
| Difficulty making decisions about how to work |  |  |  |  |  |
| Physical fatigue |  |  |  |  |  |
| Difficulty interacting with managers |  |  |  |  |  |
| Difficulty interacting with colleagues |  |  |  |  |  |
| Difficulty interacting with other healthcare professionals (such as doctors, etc.) |  |  |  |  |  |
